# A Unique Trimeric Assembly of Human Dishevelled 1 PDZ Domain in Crystal: Implication of Homo- and Hetero-Oligomerization During Wnt Signaling Process

**DOI:** 10.3390/molecules30173538

**Published:** 2025-08-29

**Authors:** Shotaro Yasukochi, Nobutaka Numoto, Kiminori Hori, Takeshi Tenno, Emi Hibino, Nobutoshi Ito, Hidekazu Hiroaki

**Affiliations:** 1 Laboratory of Structural and Molecular Pharmacology, Graduate School of Pharmaceutical Sciences, Nagoya University, Furocho, Chikusa, Nagoya, Aichi 464-8601, Japan; 2 Laboratory of Structural Biology, Institute of Science Tokyo, 1-5-45, Yushima Bunkyo-ku, Tokyo 113-8516, Japan; 3 International Center for Structural Biology, Research Institute for Interdisciplinary Science, Okayama University, Tsushima Naka 3-1-1, Kita, Okayama 700-8530, Japan; 4 BeCellBar, LLC., Kamimura-cho, Showaku, Nagoya, Aichi 466-0802, Japan; 5 Center for One Medicine Innovative Translational Research, Nagoya University, Nagoya, Aichi 464-8601, Japan

**Keywords:** PDZ domain, trimeric assembly, auto-inhibiting trimer, ligand-binding cleft, liquid–liquid phase separation, Dvl-Axin hetero-oligomerization

## Abstract

Wnt/β-catenin signaling is hyper-activated in several cancer cells and cancer stem cells. Dishevelled/Dvl is a key adapter protein that acts as a bridge between the Wnt receptor Frizzled (Fzd) and other cytosolic factors. In detail, the C-terminal cytosolic region is the ligand of the PSD-95, disks large, and zonula occludens-1 (PDZ) domain of Dvl. Therefore, the PDZ domain (Dvl-PDZ) is thought to be a potential drug target. In this paper, we determined the first crystal structure of the PDZ domain of human Dvl1 (hDvl1-PDZ) at a 2.4 Å resolution. The domain was adapted into a unique trimeric form in which all the canonical ligand-binding clefts were occupied by the β2-β3 loop of the neighbor molecule, like an auto-inhibiting trimer. We used solution nuclear magnetic resonance (NMR) experiments to assess the presence of the self-associated oligomer of hDvl1-PDZ in the solution. Introducing the Ala substitution at Asp 272, the key residue of the β2-β3 loop, partly abolished the concentration-dependent chemical shift change, which suggests that this residue is one of the key residues for formation. Based on these observations, we propose an auto-inhibiting trimer formation of Dvl-PDZ in a Dvl-Axin hetero-oligomerization model of Wnt/β-catenin signal transduction.

## 1. Introduction

The canonical Wnt signaling pathway is widely conserved in the animal kingdom and plays an important role in cell proliferation, embryonic development, differentiation, and tissue homeostasis, including the maintenance of planar cell polarity [1]. Hence, aberrant activation of this pathway often causes malformation of organs as well as cancer, whereas deficiency of the signal may cause dysgenesis and aging [2,3,4,5]. The Wnt signaling pathway transduces the extracellular stimuli from Wnt ligands into cytosolic factors via Frizzled (Fzd) receptors. The signal regulates the lifetime and the stability of the cytosolic transcription factor β-catenin, which ultimately translocates into nuclei and promotes the expression of several sets of genes [5]. However, the intermediary processes of Wnt signaling still remain unclear, especially the molecular mechanisms of adaptor proteins. Much research has demonstrated that heteromeric oligomerization of Dishevelled (Dvl) and AXIN plays a pivotal role in signaling [6,7,8]. Especially, liquid–liquid phase separation (LLPS) of Dvl and/or AXIN in cytosol, as known as “puncta”, was considered as a key feature of Wnt signaling, and historically well studied [9,10]. Recently, a glowing number of reports supported the implication of LLPS in regulation of the destruction complex [11,12,13,14,15,16,17,18]. Thus, the study for the regulation mechanisms of not only heterotypic but also homotypic oligomerization of these adaptor proteins is particularly important. 

In the absence of Wnt, cytoplasmic β-catenin is phosphorylated by a “destruction complex”, which is composed of proteins such as scaffold protein AXIN, adenomatous polyposis coli (APC), casein kinase 1, and glycogen synthase kinase (GSK) 3β. Phosphorylated β-catenin is recognized by β-TRCP, an E3 ubiquitin ligase subunit, and this triggers ubiquitin-dependent proteasomal degradation of β-catenin. On the contrary, when Wnt comes to activate Fzd and its co-receptor LRP5/6, Dvl is channeled by Fzd to the membrane using AXIN. This process is assumed to be a trigger for the formation of the Dvl-Axin hetero-oligomer [6,19]. This formation disrupts active destruction complexes to suppress the phosphorylation of β-catenin, thereby suppressing its degradation. Finally, accumulated β-catenin induces target gene expression in the nucleus.

**Figure 1:**
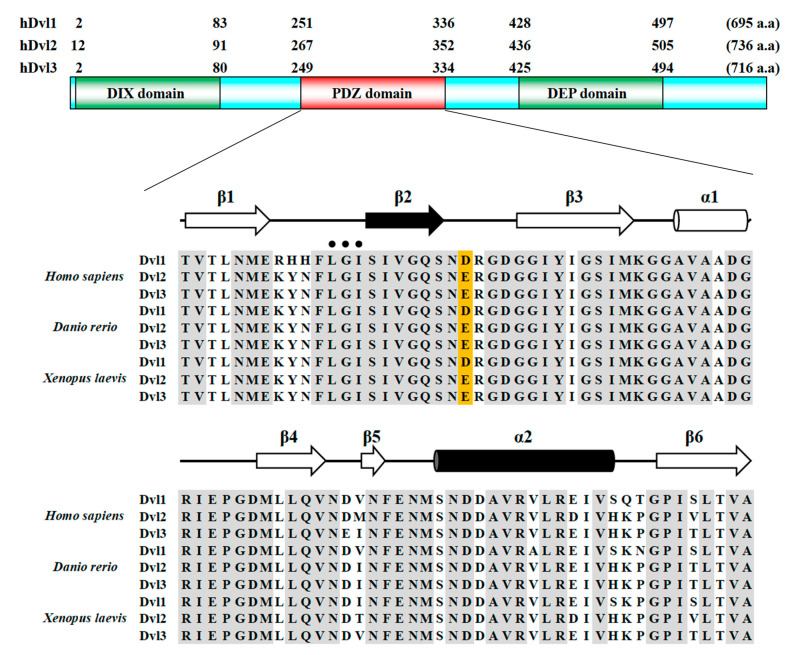
Primary structure of hDvls and the amino acid sequence alignments of the PDZ domains of Dvls from humans, zebrafish, and African clawed frogs. The conserved acidic amino acids (D or E) at the β2-β3 loop are highlighted with an orange background. The secondary structures are shown at the top of the alignments.

Dvl is a multi-domain protein composed of a DIX (Dvl/AXIN) domain [20], a PDZ (PSD-95/Dlg/ZO-1) domain [21], and a DEP (Dvl/Egl-10/Pleckstrin) domain [22] (Figure 1). In the human genome, there are three Dvl isoforms: Dvl1, Dvl2, and Dvl3 (Figure 1). It has been determined that Dvl forms not only a hetero-oligomer with AXIN during Wnt signal transduction but also forms a homo-oligomer in the absence of signal transduction. In more detail, a recent study showed that the DIX domain (Dvl-DIX) forms a helical structure in a crystal [23]. In addition, the DEP domain (Dvl-DEP) has the propensity to form a domain-swapped homodimer [24].

The detailed molecular recognition mechanism between Dvl-PDZ and the Dishevelled recognition motif (DRM) in the C-terminal region of Fzd (-KTxxxW-motif—where x is an arbitrary amino acid) in the active signaling complex had not yet been unraveled [25]. In this study, we determined the first crystal structure of the PDZ domain of human Dvl1 (hDvl1-PDZ1). The domain adapted into a unique trimeric form in which all the canonical ligand-binding clefts were occupied by the neighbor molecule, like a self-inhibiting trimer. The key residues for this association were identified. Using solution NMR, we further confirmed the presence of the self-association of hDvl1-PDZ similar to this crystal structure under high concentration conditions. The relevance of this PDZ-mediated self-assembly of Dvl in the molecular mechanism of the enigmatic Wnt/β-catenin signaling pathway is also discussed.

## 2. Results

### 2.1. Overall Structural Arrangement of the PDZ Domain of hDvl1 in Crystal 

As mentioned above, there are three isoforms of Dvl encoded in the human genome. We chose hDvl1-PDZ for this structure analysis project (Figure 1). Bacterially expressed hDvl1-PDZ in *E. coli* was purified according to our previous research with a slight modification [26].

**Figure 2:**
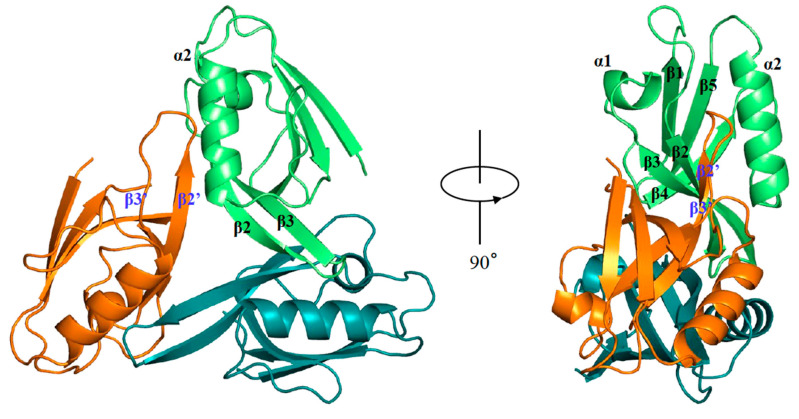
Ribbon diagram of hDvl1-PDZ trimer. Secondary structures are indicated.

hDvl1-PDZ adopted a typical structure of a PDZ domain, which consists of two α helices (α1 and α2) and five β-strands (β1, β2, β3, β4 and β5). Interestingly, an asymmetric unit contains eight protomers, and these form a unique trimeric organization in the crystal (Figure 2). Specifically, this trimer was organized into head-to-tail contact between the loop between β2 and β3 (β2-β3 loop) and the adjacent molecule. The β2-β3 loop seemed to stick to the shallow cleft between β2’ and α2’ (β2-α2 pocket) of the neighboring molecule.

### 2.2. Dvl PDZ Domain Shows Symmetrical Trimer in Crystal Structure

**Figure 3:**
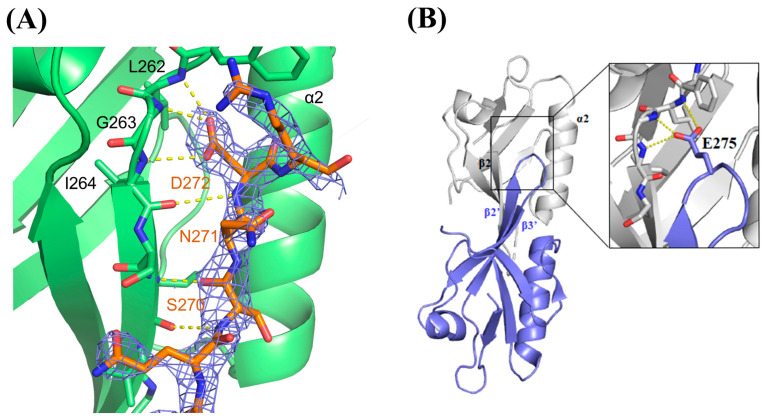
(**A**) Close-up view of the ligand-binding pocket (green). The β2-β3 loop of the adjacent protomer is represented as a stick model (orange). The electron density (2Fo-Fc) map contoured at 1.5σ of the β2-β3 loop is shown as blue mesh. The polar contacts between the protomers are indicated with a yellow dashed line. (**B**) Overall structure of xDvl2-PDZ dimer (PDB ID: 3FY5). Inset is a close-up view of the intermolecular interactions at the ligand-binding pocket. Hydrogen bonds at 275Glu (equivalent to 272Asp in hDvl1-PDZ) are indicated with a yellow dashed line.

The shallow cleft between β2 and α2 (β2-α2) is known as the canonical ligand-binding pocket, which serves as the ligand-binding site for many PDZ domains to recognize the C-terminal moieties of the target proteins. Dvl-PDZ is assumed to use the same binding cleft to recognize Fzd during signal transduction. For this reason, we hypothesized that this trimeric arrangement is a kind of the closed/inactive state, since all the Fzd binding sites are occupied and shielded by the adjacent molecules, in which the adjacent PDZ domains each fill a binding pocket. In this structure, the side chain of 272Asp at the β2 -β3 loop formed hydrogen bonds to the main chain atoms of the adjacent carboxylate binding site of hDvl1-PDZ (262Leu, 263Gly, and 264Ile). It is assumed that the carboxyl group of 272Asp mimics the carboxy terminal (COOH) group of canonical PDZ domain ligands. In addition, the three residues, 270Ser, 271Asn, and 272Asp, formed additional hydrogen bonds to the main chain atoms in β2′ of the adjacent domain (Figure 3A).

In PDB, we found that the *Xenopus* Dvl2 PDZ domain (xDvl2-PDZ) also forms a dimer in a similar self-inhibiting arrangement (Figure 3B) (PDB ID: 3FY5). This fact supported our assumption that the potential of the closed/inactive homo-oligomer (either dimer or trimer) of Dvl PDZ domains is shared by Dvl proteins when they are highly concentrated under certain cellular conditions. This assumption partially supports that those acidic amino acids (Asp or Glu), which harbor a critical carboxylic acid in their side chains to form hydrogen bonds with carboxylate binding sites, are genetically conserved in this position (Figure 1). We assume that acidic amino acids in this position may play a physiological role in forming a closed/inactive trimer form. Since the side chain of Asp is shorter than that of Glu, the β2-β3 loop can interact more closely with the adjacent protomer in the hDvl1-PDZ trimer than in the xDvl2-PDZ dimer (Figure 2 and Figure 3B). Differences in side-chain lengths between Asp and Glu might contribute to whether they form the trimer or dimer.

### 2.3. Equilibrium of hDvl1 PDZ Self-Assembly in Solution Confirmed by NMR and Dynamic Light Scattering Experiments

**Figure 4:**
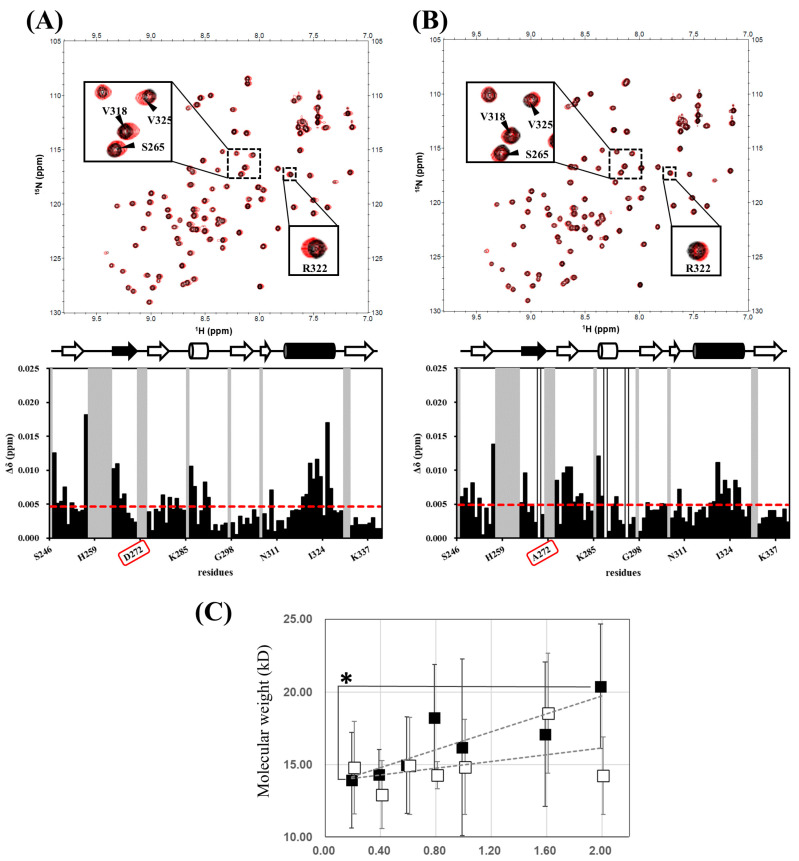
Assessment of self-oligomerization of hDvl1-PDZ by solution NMR method. ^1^H–^15^N HSQC spectra of 1.0 mM protein (black) was overlayed on that of 0.1 mM protein (red) (top). Normalized chemical shift changes (Δ*δ*) were plotted against each residue (bottom). (**A**) Wild type. (**B**) D272A mutant. Schematic drawing of the secondary structure of hDvl1-PDZ is also shown in the figure. (**C**) Concentration dependent equilibrium monitored by dynamic light scattering. Filled square, wild type, open square, D272A mutant. Standard deviations were indicated by error bars. Dashed lines represent linear approximations. * indicates statistical significance *p* < 0.05.

Although we observed a unique intermolecular trimeric arrangement of hDvl1-PDZ that is likely a closed/inactive conformation, we further assessed the existence of the trimer under a physiological solution condition. For this purpose, we collected ^1^H–^15^N HSQC spectra under low (0.1 mM) and high (1.0 mM) protein concentration conditions in order to detect any signal changes upon equilibrium shift between monomeric and oligomeric states. Although the NMR concentration dependence experiment is unable to discriminate the oligomerization number of monomer-oligomer equilibrium, it should be noted that the NMR experiment is still useful for assessing either presence or absence of monomer-oligomer equilibrium in ‘undefined’ number of oligomeric assemblies. When comparing the two spectra, small but definite chemical shift changes were observed in more than ten amide signals (Figure 4A). As we expected from the crystal structure, these altered signals were derived from the residues surrounding the ligand-binding cleft (β2-α2).

This result partially supports the conclusion that hDvl1-PDZ potentially forms a self-associating oligomer not only in the crystal but also in a solution at a relatively high concentration such as in LLPS. As mentioned before, we were unable to determine whether the self-assembly was a trimer or not, because the signal change was not large enough for further quantitative analysis. For the same reason, we were unable to determine the *K*_d_ for this self-assembly. Given this, we generated a D272A mutant of hDvl1-PDZ in which the 272Asp located in the β2-β3 loop was substituted with alanine. The residue forms a hydrogen bond with the carboxylate binding site of hDvl1-PDZ in the crystal (Figure 3A). We measured ^1^H–^15^N HSQC (Figure 4B) again and then observed certain chemical shifts in the corresponding spectra. The signal changes (~70% smaller than those of WT) observed at residues surrounding the carboxylate binding pocket in D272A support our hypothesis of the potential of self-inhibiting oligomer model of hDvl1-PDZ1 was confirmed. Accordingly, the acidic amino acid side chain of 272Asp may partially contribute to stabilizing the closed/inactive state by forming a hydrogen bond to the carboxylate binding site.

Finally, we further examined the self-assembly of hDvl1 PDZ in solution using an alternative method: dynamic light scattering (DLS) (Figure 4C), the statistical analysis of DLS analyses is shown in Table S2. As the protein concentration increased from 0.2 mM to 2.0 mM, we observed a gradual rise in the apparent molecular weight of hDvl1 PDZ. While the statistical differences in these wildtype data were generally not significant, the data at 2.0 mM against 0.2 mM was below 0.05. At the highest concentration tested (2.0 mM), the wildtype hDvl1 PDZ exhibited an apparent molecular weight of approximately 20 kD (the calculated molecular weight of the monomer is 10,754), suggesting an equilibrium between monomeric and dimeric states. However, as this upward trend did not plateau at 2.0 mM, the involvement of higher-order oligomers—such as trimers—cannot be excluded based on this data. In contrast, the D272A mutant displayed only a modest concentration-dependent increase in molecular weight. These modest increased molecular weights did not show statistical differences and are consistent with the reduced self-association observed in the preceding NMR experiments.

## 3. Discussion

While activated Wnt signaling in cells, self-assembly and oligomer formation of Dvl is believed to have certain physiological roles. There is evidence suggesting that fluorescent-tagged Dvl forms oligomers, and it has been observed as foci upon Wnt/β-catenin signal activation [6,19,20,27,28]. It was also reported that the signal transduction was not completed without these oligomer formations. In 2007, Bienz et al. showed that the DIX domain of Dvl (DIX) plays an important role in this oligomerization with the DIX domain of the AXIN (DAX) protein [19]. DIX and DAX formed heteromeric helical oligomers in the crystal structure. In 2015, they revealed that the DIX domain of hDvl2 also formed helical oligomers on its own in the crystal structure [23]. Furthermore, in 2016, she and her colleagues reported that the DEP domain of Dvl formed a unique domain-swapping dimer in the crystal. They also demonstrated that this dimerization was indispensable for Wnt/β-catenin signaling [24]. 

**Figure 5:**
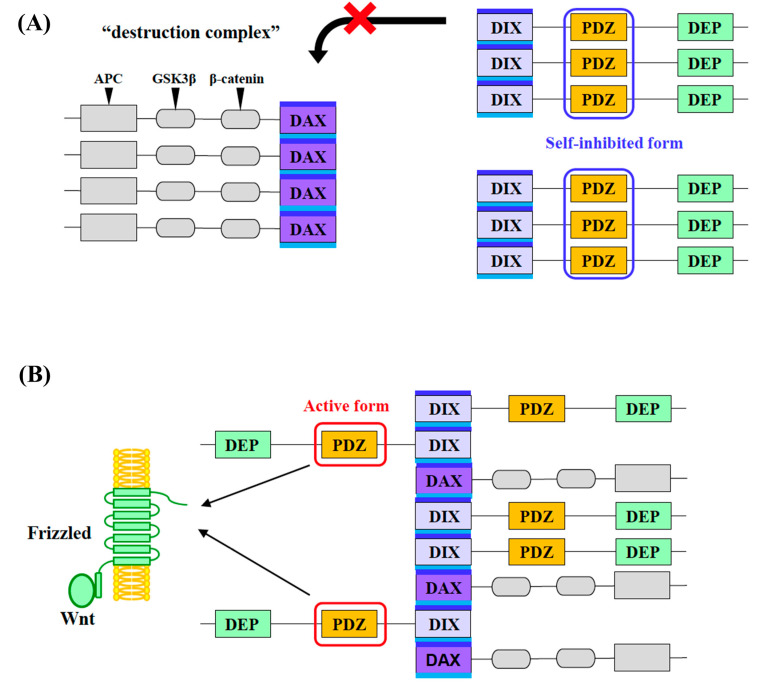
Schematic diagram of signal transduction of Wnt by rearrangement of homo-oligomers of AXIN and Dvl (**A**) into hetero-oligomer of AXIN/Dvl triggered by Fzd-Dvl interaction in the presence of Wnt ligand (**B**). (**A**) AXIN is self-assembling and forms a homo-oligomer through oligomerization of the DAX domain, while the oligomer recruits catenin, GSK3, and APC to maintain the destruction complex (left). Meanwhile, Dvl oligomerizes mainly through the DIX domain. However, the DEP domain is also able to form dimers, and the PDZ domain can form trimers, as demonstrated in this research. The trimeric form of PDZ is self-inhibited and thereby unable to bind Fzd. (**B**) Upon Wnt ligand binding, interaction between the C-terminal region of Fzd and Dvl is initiated. This may induce the rearrangement of two homo-oligomers, Dvl and AXIN, into heteromeric oligomers. Accordingly, the destruction complex is disassembled and becomes inactive.

Taking all these previous studies into account, we hypothesize that Dvl is likely to form a homo-oligomer through its multiple inter-domain interaction in cells. If so, it is also likely that the local concentration of the PDZ domain in the Dvl molecule would be increased upon oligomer formation. We assume that the closed/inactive trimer or oligomer structure of Dvl-PDZ possibly exists in the self-oligomerized Dvl molecules. In contrast, Dvl-PDZ is known to play an essential role as the binding interface to the cytoplasmic region of the Fzd, Wnt receptor. Thus, the ligand-binding site of Dvl-PDZ must be unoccupied when a Wnt signal is coming through Fzd. At the same time, it is also known that AXIN must be involved in the signaling complex formed by Fzd and Dvl when the signal is activated. Although the molecular mechanism of Dvl and AXIN in activated Wnt signaling has not yet been examined, it is believed that hetero-oligomerization between DIX-DAX and homo-oligomerization of Dvl play key roles in this process. In this study, we propose a new model for a Wnt signal transduction mechanism involving the equilibrium shift between two distinct biomolecular condensates, that are likely regulated by self-assembly of Dvl-PDZ domains (Figure 5). This model is consistent with our observation that hDvl1-PDZ adopted homo-trimer in the crystal structure. 

In our model, Dvl is in a closed/inactive homo-oligomer in the absence of the Wnt ligand, while AXIN forms a homo-oligomer via DAX. As a result, several key factors, such as β-catenin and GSK3β, are recruited by the AXIN oligomer, thereby forming a “destruction complex” to accelerate the degradation of β-catenin. It was reported that the affinity between DAX-DIX is weaker than that of the homo-oligomeric assembly of DAX–DAX. Thus, during the formation of a destruction complex, we assume that cytosolic Dvl cannot interrupt the AXIN oligomer spontaneously. The closed/inactive oligomer formation of Dvl PDZ is likely to maintain this state. 

The proposed trimeric auto-inhibition of Dvl1-PDZ (Figure 5) represents an intermolecular regulatory mechanism. However, this model must be integrated with established intramolecular auto-inhibition, wherein the C-terminal region of Dvl binds to its own PDZ domain, forming a closed conformation that sterically blocks ligand binding. This intramolecular mechanism, validated by Qi et al. [29], operates via high-affinity interactions (Kd = ~7 μM) between the Dvl C-terminus and the PDZ ligand-binding cleft, effectively preventing Frizzled engagement in the absence of Wnt. Structural studies [30] confirm that C-terminal deletion mutants disrupt this auto-inhibition, enhancing non-canonical Wnt signaling. Crucially, this intramolecular “lock” functions independently of concentration, providing baseline inhibition at low Dvl concentrations.

Under conditions of elevated local Dvl concentration such as small puncta formation observed before Wnt-stimulation in certain cancer cell lines [11,31], the intramolecular auto-inhibition is supplemented by the concentration-dependent intermolecular trimerization observed in our NMR data (Figure 4A). It is noteworthy that enrichment factors for proteins within biomolecular condensates formed via LLPS are frequently reported to be around 100-fold [32]. In parallel, in vitro studies of the isolated Dvl2-DIX domain have demonstrated that phase separation initiates at a minimum concentration of 12 μM [33]. Based on these observations, we estimate that the local concentration of Dvl within puncta exceeds 1000 μM. This concentration range is sufficient to promote the intermolecular PDZ–PDZ interactions characterized in this study. This trimeric assembly reinforces auto-inhibition through: 

(i) Steric blocking: The β2-β3 loop of one protomer occupies the ligand-binding cleft of an adjacent protomer (Figure 3A), mimicking the intramolecular C-terminal inhibition but with higher avidity due to multivalency.

(ii) Asp272-mediated stabilization: Hydrogen bonds involving Asp272 (Figure 3A) rigidify the trimer interface, creating a cooperative energy barrier against dissociation. This dual-layered regulation ensures that high Dvl concentrations typical in signalosomes amplify auto-inhibition, preventing premature pathway activation. 

Note that the existing intramolecular model and our trimeric mechanisms are not mutually exclusive each other; they may operate synergistically to enforce a “conformational hierarchy” as follows. When the Dvl concentration is low, intramolecular C-terminal binding may dominate, thereby maintaining a monomeric closed state. However, if Dvl concentrations turn into high, trimerization may take precedence, leveraging local density to stabilize a self-inhibited oligomer (Figure 5B). Disruption of both mechanisms via Wnt-induced Frizzled binding releases the PDZ cleft for downstream interactions (e.g., Dvl-Axin hetero-oligomerization).

Meanwhile, in the presence of the Wnt ligand, the ligand-activated Fzd may change its conformation to recruit Dvl, and then Dvl is localized around the plasma membrane and concentrated. This increases the local concentration of Dvl and may trigger Dvl-DIX to be inserted into the DAX oligomer, and LLPS of Dvl/AXIN heterotypic oligomers is promoted by multivalent protein–protein interactions among them, followed by the rearrangement from the homo-oligomers (DIX and DAX) into the hetero-oligomers. When Dvl and AXIN form hetero-oligomers, the dissociation of the Dvl-PDZ trimer and the exposure of Fzd-binding pocket of Dvl-PDZ may occur concertedly. At the same time, the formation of the Dvl-Axin hetero-oligomers could lead to the collapse of the AXIN-centered destruction complex, probably via equilibrium shift in the phase-separated homotypic and heterotypic BMCs, because the local concentration of AXIN is reduced in the hetero-oligomers. All of these processes are probably concerted. Finally, the degradation of β-catenin is suppressed (Figure 5). We summarize this concentration-dependent shift in self-inhibition mechanisms into Table 1.

In conclusion, we succeeded in determining the crystal structure of the PDZ domain in hDvl1 in a unique self-assembling trimeric arrangement. In the crystal, the β2-β3 loop of hDvl1-PDZ contacted the canonical binding cleft. The sidechain of a well conserved aspartic acid (272Asp) mimicked the carboxy terminal COOH group of the canonical PDZ ligands and was occupying the binding pocket, thus the interaction between Fzd and hDvl1-PDZ seemed to be inhibited. The molecular contacts found in the interaction are potentially useful for designing non-peptidic inhibitors against Dvl1-PDZ. An NMR experiment partly demonstrated that this self-assembly existed in a solution. Based on these observations, we propose that the self-inhibiting assembly of Dvl1-PDZ can function as a stabilizing mechanism for the resting Dvl homo-oligomer in the Wnt/β-catenin signal transduction process.

## 4. Materials and Methods

### 4.1. Preparation of Protein Samples 

The expression and purification of the human Dvl1-PDZ (residues 246–340) with two amino acid substitutions (CW338, 339AT) was previously described [26]. The plasmid carrying the mutant D272A, hDvl1-PDZ(D272A) was prepared according to a standard PCR mutagenesis method. In brief, the recombinant glutathione-S-transferase (GST)-tagged form of human Dvl1-PDZ and its mutant were expressed by *Escherichia coli* BL21 (*DE3*) in 1 L LB (Nacalai Tesque Inc., Kyoto, Japan) or M9 minimal media with ^15^N-ammonium chloride (Cambridge Isotope Laboratories, Tewksbury, MA, USA, if needed) as the sole nitrogen source, supplemented with divalent cations and vitamins at 37 °C. The protein expression was induced by the addition of final 0.2 mM of isopropyl-β-D-galactoside (IPTG, Nacalai Tesque), with immediate lowering of the temperature to 20 °C. The cells were harvested 20 hr after IPTG induction. The fusion protein was then affinity-purified using resin (GST-Accept™; Nacalai Tesque). The GST tag was removed by PreScission protease on beads. The protein solution was loaded on a Superdex 75 HR 26/60 column (GE Healthcare, Little Chalfont, UK) equilibrated with 50 mM Tris-HCl (pH 7.2) and 150 mM NaCl (Nacalai Tesque).

### 4.2. Crystallization, Data Collection, Structure Determination, and Refinement 

For the crystallization of hDvl1-PDZ, the purified protein in the buffer containing 0.01 M Tris-HCl pH 7.5, 0.025 M NaCl was concentrated to 8–9.5 mg/ml. The crystallization conditions were screened based on the sparse matrix method using commercially available screening kits (Crystal Screen, Crystal Screen 2, and Additive Screen Kits, Hampton Research, Aliso Viejo, CA, USA) with the hanging-drop or sitting-drop vapor-diffusion method at 20 °C. The best crystals were obtained from the crystallization solution containing 0.1 M sodium citrate tribasic dehydrate pH 5.6, 0.5 M ammonium sulfate, 1.0 M lithium sulfate monohydrate, 0.01 M glutathione, and 0.01 M glutathione disulfide (Hampton Research). Prior to data collection, all the crystals were soaked in cryo-protectant solutions containing 20% (*v*/*v*) glycerol (Nacalai Tesque), along with their respective crystallization solutions, and were flash-frozen using liquid nitrogen.

X-ray diffraction experiments were performed at beamlines NW12A at KEK PF-AR and BL38B1 at SPring-8 [34]. All data were processed and scaled using XDS [35] and truncated using the CCP4 software suite [36]. For further refinement, 5% of the reflections were randomly and independently selected as a test set to calculate the R_free_ values.

The structures of *Xenopus laevis* Dvl PDZ (PDB ID: 3FY5) were used as search models to determine the initial phases with the molecular replacement method using PHASER (2.5.0) [37]. Several cycles of manual model rebuilding and refinement were conducted using COOT (1.1.10) [38] and PHENIX (1.20) [39], respectively. The refined models were validated with MolProbity (4.3) [40]. The statistics of the data collection and refinement are summarized in Table S1. The figures were prepared using PYMOL graphic software (http://www.pymol.org/ (accessed on 20 July 2023)).

The coordinates and structural data have been deposited at the Protein Data Bank Japan (PDB ID: 6LCA, https://pdbj.org/mine/summary/6lca (accessed on 22 November 2023))—a member of the Worldwide Protein Data Bank.

### 4.3. NMR Experiments

For this study, two-dimensional NMR spectra were measured using NMR spectrometer (900 MHz, Bruker Avance III; Bruker Analytik GmbH, Karlsruhe, Germany) equipped with a cryogenic triple-resonance probe. For NMR self-association experiments, 1.0 mM, and 0.1 mM PDZ domain sample were dissolved in 250 μL of 90 mM potassium phosphate buffer (pH 7.4, Nacalai Tesque) containing 0.45 mM EDTA (Nacalai Tesque) supplemented with 10% D_2_O (Cambridge Isotope Laboratories). Then, the ^1^H–^15^N heteronuclear single quantum coherence (HSQC) spectra were obtained. All NMR spectra were recorded at 293 K. All spectra were processed using NMRPipe (11.4) and were analyzed using the program Sparky version 3.114 (http://www.cgl.ucsf.edu/home/sparky/, accessed on 10 May 2022). The backbone signal assignment was according to our previous report [26]. All chemical shift changes in the ^1^H–^15^N HSQC spectra were calculated as Δ*δ*_normalized_ = {Δ*δ*(^1^H)^2^ + [Δ*δ*(^15^N)/6]^2^}^1/2^. The chemical shift changes were then mapped onto the corresponding residues of the structure of hDvl1-PDZ using PYMOL.

### 4.4. Dynamic Light Scattering

Dynamic light scattering (DLS) experiments were conducted using a Zetasizer μV instrument (Malvern Instruments, Malvern, UK), employing 1.25 mm square quartz cuvettes (105.231-QS, Hellma GmbH & Co. KG, Müllheim, Germany). The solution containing wildtype and D272A mutant hDvl1-PDZ were prepared to a concentration of 0.2, 0.4, 0.6, 0.8, 1.0, 1.6 and 2.0 mM in 100 mM potassium phosphate buffer (pH 7.4) containing 0.5 mM EDTA. The built-in size determination mode of the instrument was employed, which estimated the particles sizes of one small and two large particles in the sample solution with their abundance. The estimated molecular weight of the most abundant particle (usually > 97%) is used for further concentration dependency analysis. Each analysis was repeated six sub-runs at 298 K and an equilibration period of 120 s. Statistical differences were determined by Dunnett’s multiple comparison test performed using R (version 4.4.3).

## Figures and Tables

**Table 1:** Comparison of two alternative self-inhibition mechanisms of Dvl proteins.

	Intramolecular	Intermolecular
Interaction Type	PDZ: C-terminus	PDZ-PDZ
PDZ Binding Mode	canonical	non-canonical
Cellular Location	cytosol	puncta
Effective Concentration	below 12 µM	>1000 µM
Regulatory Trigger	post-translational modification	elevated concentration
Reference	Qi et. al. [29]	this study

## Data Availability

The datasets analyzed for this study can be found in the Protein Data Bank (PDB code, 6LCA) [https://www.rcsb.org/structure/6LCA (accessed on 22 November 2023)]. The NMR dataset is deposited to Nagoya Repository [https://nagoya.repo.nii.ac.jp/records/2013187 (accessed on 21 August 2025)].

## Supplementary Materials

The following supporting information can be downloaded at: https://www.mdpi.com/article/10.3390/molecules30173538/s1, Table S1: Data Collection and Refinement Statistics, Table S2: Statistical analysis of DLS analyses.

## Abbreviations

The following abbreviations are used in this manuscript:

PDB	Protein Data Bank
Dvl	Dishevelled
PDZ	PSD-95, disks large, and zonula occludens-1
DIX	Dvl/AXIN
DEP	Dvl/Egl-10/Pleckstrin
Fzd	Frizzled
LLPS	Liquid–liquid phase separation
NMR	Nuclear magnetic resonance
HSQC	Heteronuclear single quantum coherence spectroscopy
GSK	glycogen synthase kinase
APC	adenomatous polyposis coli
