# Supplementary material for: A Unique Trimeric Assembly of Human Dishevelled 1 PDZ Domain in Crystal: Implication of Homo- and Hetero-Oligomerization During Wnt Signaling Process
Source: Molecules. 2025 Aug 29;30(17):3538. doi: 10.3390/molecules30173538 (PMC12430135; doi:10.3390/molecules30173538)
Supplement: Supplementary file 1 [file molecules-30-03538-s001.zip › molecules-3762729-supplementary.pdf]

Supplementary materials

A Unique Trimeric Assembly of Human Dishevelled 1 PDZ Domain in Crystal: Implication of Homo- and Hetero-Oligomerization During Wnt Signaling Process

Shotaro Yasukochi [1,†], Nobutaka Numoto [2,3,†], Kiminori Hori [1], Takeshi Tenno [1,4], Emi Hibino [1], Nobutoshi Ito [2,*] and Hidekazu Hiroaki [1,4,5,*]

[1] Laboratory of Structural and Molecular Pharmacology, Graduate School of Pharmaceutical Sciences, Nagoya University, Furocho, Chikusa, Nagoya 464-8601, Aichi, Japan
[2] Laboratory of Structural Biology, Institute of Science Tokyo, 1-5-45, Yushima Bunkyo-ku, Tokyo 113-8516, Japan
[3] International Center for Structural Biology, Research Institute for Interdisciplinary Science, Okayama University, Tsushima Naka 3-1-1, Kita, Okayama 700-8530, Japan
[4] BeCellBar, LLC., Kamimura-cho, Showaku, Nagoya, Aichi 466-0802, Japan
[5] Center for One Medicine Innovative Translational Research, Nagoya University, Nagoya 464-8601, Aichi, Japan
* Correspondence: ito.str@tmd.ac.jp (N.I.); hiroaki.hidekazu.j7@f.mail.nagoya-u.ac.jp (H.H.); Tel.: +81-3-5803-4594 (N.I.); +81-5-2789-4535 (H.H.)
† These authors contributed equally to this work.

Supplementary Table S1. Data Collection and Refinement Statistics

**Data Collection**	
Space group	*H*3
Unit cell parameters (Å)	*a* = 147.9, *c* = 79.0
Wavelength (Å)	1.0000
Resolution range (Å)	30-2.40 (2.55-2.40)
Total number of unique reflections	25,203
Completeness (%)	99.9 (98.8)
Average $I/\sigma(I)$	14.3 (1.7)
Redundancy	5.0 (5.2)
R_{sym}	0.063 (0.774)
$CC_{1/2}$ (%)	99.9 (74.3)
**Refinement**	
R_{work}/R_{free}	0.245/0.273
Number of atoms	
Protein	5,368
Sulfate ion	40
Water	22
Average *B* factor (Å^2)	66.2
Root mean square deviations	
Bond lengths (Å)	0.002
Bond angles (°)	0.54
Ramachandran analysis	
Favored (%)	98.4
Allowed (%)	1.6
Outliers (%)	0

Values in parentheses are for the highest-resolution shell.

Supplementary Table S2. The results and the statistical analysis of DLS experiments.

Apparent molecular weight (kDa) measured by DLS							
**wildtype**	concentration (mM)						
run							
	0.20	0.40	0.60	0.80	1.00	1.60	2.00
#1	20.20	15.70	13.00	21.50	19.60	16.70	13.20
#2	12.00	12.50	11.30	13.40	13.00	15.90	17.90
#3	13.50	15.20	17.50	20.00	11.80	14.20	22.80
#4	11.10	15.90	14.50	22.60	27.20	17.20	22.40
#5	12.20	11.80	13.20	16.60	12.80	26.50	20.90
#6	14.60	14.80	20.30	15.30	12.80	12.10	25.20
AVERAGE	13.93	14.32	14.97	18.23	16.20	17.10	20.40
SD	3.31	1.74	3.33	3.67	6.09	4.97	4.27
**D237A mutant**							
run							
#1	14.10	10.20	15.50	15.40	12.50	15.20	12.20
#2	18.10	12.40	14.50	13.60	12.70	24.10	14.50
#3	12.10	16.50	20.80	14.50	14.10	15.50	16.10
#4	11.20	12.30	10.50	13.00	14.60	23.50	13.40
#5	19.10	14.90	13.80	14.00	21.30	16.00	11.00
#6	14.20	11.30	14.50	15.20	13.90	16.90	18.30
AVERAGE	14.80	12.93	14.93	14.28	14.85	18.53	14.25
SD	3.18	2.34	3.35	0.93	3.26	4.12	2.66

Statistical analysis				
	diff	lwr.ci	upr.ci	pval
**wildtype**				
0.4-0.2	0.3833333	-6.02670788	6.793375	1.0000
0.6-0.2	1.0333333	-5.37670788	7.443375	0.9957
0.8-0.2	4.3000000	-2.11004121	10.710041	0.2978
1.0-0.2	2.2666667	-4.14337455	8.676708	0.8466
1.6-0.2	3.1666667	-3.24337455	9.576708	0.5935
2.0-0.2	6.4666667	0.05662545	12.876708	**0.0473 ***
**D237A mutant**				
0.4-0.2	-1.8666667	-6.5178253	2.784492	0.7676
0.6-0.2	0.1333333	-4.5178253	4.784492	1.0000
0.8-0.2	-0.5166667	-5.1678253	4.134492	0.9994
1.0-0.2	0.0500000	-4.6011586	4.701159	1.0000
1.6-0.2	3.7333333	-0.9178253	8.384492	0.1557
2.0-0.2	-0.5500000	-5.2011586	4.101159	0.9992

(1) Dunnett's test for comparing several treatments with a control (0.2): 95% family-wise confidence level.
(2) Signif. codes: '***' , 0.001, '**' , 0.01, '*' , 0.05, '-' , 0.1
